# Freeze–Thaw Damage Degradation Model and Life Prediction of Air-Entrained Concrete in Multi-Year Permafrost Zone

**DOI:** 10.3390/ma16247703

**Published:** 2023-12-18

**Authors:** Kai Zhang, Aojun Guo, Yonghui Yu, Bo Yang, Bentian Yu, Chao Xie

**Affiliations:** 1 Civil Engineering Department, Lanzhou Jiaotong University, Lanzhou 730070, China; zhangkai0212@yeah.net (K.Z.); yyh1056848662@163.com (Y.Y.); yangbo4892@yeah.net (B.Y.); yubentian@mail.lzjtu.cn (B.Y.); zhanghl000210@163.com (C.X.); 2 National and Provincial Joint Engineering Laboratory of Road & Bridge Disaster Prevention and Control, Lanzhou Jiaotong University, Lanzhou 730070, China; 3 Northwest Institute of Eco-Environment and Resources, Chinese Academy of Sciences, Lanzhou 730000, China

**Keywords:** negative-temperature environment, air-entrained concrete, pore structure, freeze–thaw damage degradation model, life prediction

## Abstract

The Qinghai–Tibet Plateau is the main permafrost area in China. Concrete structures constructed on permafrost are affected by the early negative-temperature environment. In particular, the negative-temperature environment seriously affects the strength growth process and the frost resistance of concrete (FRC). Therefore, this study considered the influence of the gas content, water–binder ratio (w/b), age, and other factors on the strength variation law and FRC under −3 °C curing conditions. Nuclear magnetic resonance (NMR) was used to analyze the pore structure of concrete before and after freeze–thaw cycles (FTCs). The results showed that the compressive strength of the concrete (CSC) under −3 °C curing was only 57.8–86.4% of that cured under standard conditions. The CSC under −3 °C curing showed an obvious age-lag phenomenon. The FRC under −3 °C curing was much lower than that under standard curing. The porosity of the concrete under −3 °C curing was greater, with a higher percentage of harmful and multi-harmful pores than that under standard curing. The concrete properties deteriorated primarily because curing at −3 °C hindered the hydration reaction compared with standard methods. This hindrance resulted in diminished hydration development, weakening the concrete’s structural integrity. Under both curing conditions, when the gas content was between 3.2% and 3.8%, the frost resistance was the best. This is because a gas content within this range effectively enhances the internal pore structure, therefore relieving the swelling pressure caused by FTCs. Based on the freeze–thaw damage (FTD) model proposed by previous authors, a new model for the CSC under −3 °C curing reaching that of the concrete under standard curing for 28 d was established in this study. This advanced model was capable of accurately assessing the FTD of concrete structures in permafrost regions. Finally, the life expectancy of concrete in Northwest China was predicted. The life of the concrete reached 46.9 years under standard curing, while the longest life of the concrete under −3 °C curing was only 12.9 years. Therefore, attention should be paid to constructing and curing concrete structures in cold environments.

## 1. Introduction

Generally speaking, frozen soil comprises a variety of rocks and soil types at temperatures below 0 °C containing ice. Depending on its freezing state and duration, frozen soil can be divided into short-term frozen soil, seasonally frozen soil, and permafrost, which can be further divided into unstable permafrost (above −0.5 °C), sub-stable permafrost (−0.5 °C to −3 °C), and stable permafrost (below −3 °C) according to the average annual ground temperature. The area of permafrost in China is approximately 2.15 million km^2^, accounting for 22.3% of the national land area [1]. The southern Qinghai and northern Tibet regions are areas of developing permafrost. These two areas are traversed by the Sichuan–Tibet railway, which is under construction, and the Qinghai–Tibet highway, which is yet to be built. The permafrost development areas in this region are either distributed continuously or over large areas. In addition, the temperature in this area is low; the underground frozen layer is thick, and the average annual ground temperature along the route is in the range of 0 °C to −4 °C [2,3].

Permafrost is a soil medium that is extremely sensitive to temperature and external factors. Influenced by concrete construction, heat, and exothermic hydration, its spatial variability and thermal disturbance cause the physical field and upper limit of permafrost to change in the permafrost zone. This affects the thermal stability of the permafrost and accelerates the process of permafrost degradation [4,5,6]. Therefore, the concrete of the cast-in piles constructed in the frozen soil area of the Qinghai–Tibet Plateau is affected by temperature in the early stages. This leads to a decrease in the strength and durability of the concrete [7]. Insufficient frost resistance in the later stages is the main reason for the failure of concrete durability [8]. This can directly affect the stability and safety of bridge and culvert structures, leading to permanent defects in concrete structures in the frozen soil layer. It could even endanger the safe operation of the railroad [9,10,11].

Many scholars have researched the freeze–thaw damage (FTD) mechanism of concrete. Wang et al. [12] described three stages of FTD from a microscopic perspective: water absorption, freezing, and structural damage. Powers [13] analyzed the influence of pore water on concrete pore walls from a submicroscopic perspective and proposed the theory of hydrostatic pressure. It was concluded that FTD was caused by hydrostatic pressure due to the freezing of water in the capillary pores. Later, Powers and Helmuth [14] proposed the osmotic pressure theory, which suggests that FTD is mainly caused by the difference in osmotic pressure between frozen pores and unfrozen gel pores. The FTD process of concrete is irreversible and can be regarded as a fatigue damage process.

Deng et al. [15] studied the damage process and mechanism of air-entraining regenerated concrete under a freeze–thaw environment from microscopic aspects. Their results showed that the addition of air-entraining agents could improve the pore structure and frost resistance of concrete (FRC). The air-entraining agent used was a water-repellent active agent. When such an agent is added to the concrete mix, many tiny bubbles can be produced in the mixing process. These bubbles can improve the durability of the concrete and ensure that some basic mechanical properties of the concrete are not damaged [16,17,18,19].

Piasta [20] found that the incorporation of air-entraining agents could effectively improve the FRC by comparing the salt-freezing resistance of ordinary silicate cement mortar with and without air-entraining agents. Zheng [21] found that concrete mixed with a certain amount of an air-entraining agent significantly improved the FRC, and the flexural strength reached its maximum when the gas content was 4%. AL-Assadi [22] studied the mechanical properties of concrete mixed with an air-entraining agent using the FTC test. They found that after freeze–thaw cycles (FTCs), the concrete had better mechanical properties than the concrete without using an air-entraining agent. Zhang [23] analyzed the effects of gas content on frost resistance from the perspective of pore structure. They found that when the gas content ranged from 2% to 6%, the percentage of tiny closed pores in the concrete increased significantly, and the frost resistance increased with the increase in gas content. Recent research has focused on concrete materials and structures subjected to standard curing. However, the following aspects have not been systematically studied: whether the macroscopic properties of concrete under standard curing, as defined by conventional engineering, are fully compatible with the current engineering conditions for construction in multi-year frozen soils and whether the development of FRC is affected by freezing damage.

Yu et al. [24] formulated FTD equations for concrete in both water and salt freeze–thaw conditions. Their approach integrated fatigue damage accumulation theory with the Aas–Jakobsen S–N formulation. Similarly, Bai et al. [25] adapted fatigue damage theory to create macro and micro FTD equations specific to aeolian sand concrete, drawing on principles of irreversible thermodynamics and continuous damage mechanics. Although these models focus on concrete under standard curing conditions, there is a notable gap in research for FTD models under negative-temperature curing. Additionally, most previous models did not account for the effects of gas content on FTD processes. However, recognizing the relationship between gas content and compressive strength opens avenues for developing versatile FTD models applicable under diverse curing conditions.

In this study, extensive compressive strength and FTC tests were conducted on concrete with varying gas content levels to evaluate their impact on the material’s properties. These tests included comparisons between −3 °C and standard curing conditions, highlighting differences in outcomes. The study particularly focused on the effects of −3 °C curing on the compressive strength of concrete (CSC) and FRC. Building upon existing FTD models, a new model (FTD model) was developed, specifically designed to factor in the strength under −3 °C curing conditions. This model has proven highly effective in accurately predicting FTD in concrete subjected to −3 °C curing.

In this study, concrete specimens with varying water–binder ratios (w/b) and gas contents were prepared to explore their behavior under different conditions. These specimens underwent curing at both −3 °C and standard temperatures, with the selection of −3 °C based on its critical role as a threshold in frozen soil states. The tests included both compressive strength and FTC analyses. This research primarily assessed how gas content, curing age, and w/b influence compressive strength, as detailed in Section 3.1. The study also examined the effect of gas content and w/b on FRC from macroscopic and microscopic viewpoints, with further information available in Section 3.2. Building on findings from previous FTD models and freeze–thaw tests, a new FTD model was formulated based on the equal strength theory, specifically for concrete cured at −3 °C. The study also included an analysis to predict the lifespan of concrete in Northwest China, focusing on understanding the variations in strength and behavior of FRC under −3 °C curing conditions, thus providing insights for concrete construction in cold regions.

## 2. Material and Methods

### 2.1. Raw Materials

This study used Gansu Qilian Mountain ordinary Portland cement (P.O 42.5), with specifications in Table 1. The mineral admixture, a mix of equal parts II-grade fly ash and S95-grade mineral powder from Gansu, China, was utilized. River sand, detailed in Table 2, served as the fine aggregate, featuring a 2.2 fineness modulus and 2620 kg/m^3^ density. The coarse aggregate, contrasting the fine, was rough-surfaced, hard-textured crushed stone, with particle sizes of 5–31.5 mm and a density of 2670 kg/m^3^. External additives included a China Sobute air-entraining agent and a 26.5% effective retarding polycarboxylic acid-based water-reducing agent (Sobute New Materials Co., Ltd., Nanjing, Jiangsu, China). Tap water was used for concrete mixing.

### 2.2. Specimen Preparation and Mix Proportions

In this test, conforming to JGJ 55–2011 standards [26], concrete mixes with w/b of 0.38, 0.31, and 0.24 were prepared to examine the effects of gas content on CSC and FRC. To achieve this, an air-entraining agent was added, resulting in gas contents in the concrete varying from 0.8% to 9.6% [27]. This variable is referred to as ‘gas content’ throughout this article. Measuring gas content involved mixing the concrete, placing it into a cylinder, and using vibration to determine the content until no large bubbles remained on the surface. The specific mix compositions are listed in Table 3. Workability was maintained by keeping the slump and extension within 180–200 mm and 500–550 mm, respectively, adjusting the amount of water-reducing agent as needed. Our research indicated that a 15% dosage of both fly ash and mineral powder yielded the best mechanical properties and durability [28], which was the selected dosage for these admixtures.

### 2.3. Curing Environmental Conditions

**Figure 1:**
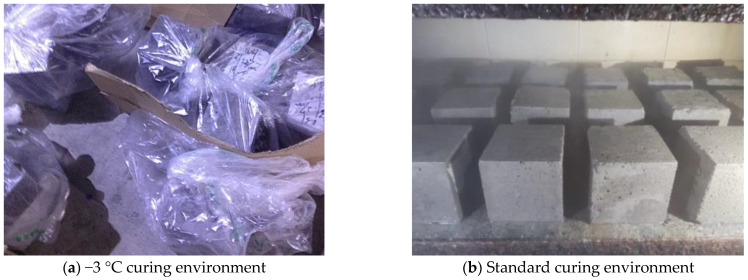
Concrete curing method.

In this study, concrete specimens were cured according to the GB/T50080–2016 standard [29]. After casting, a portion of the specimens was placed in an atmosphere simulation box, set at −3 ± 0.2 °C and over 95% humidity, for mold curing. To address the difficulty of maintaining high humidity at sub-zero temperatures, each specimen was bagged before placement in the box. These were demolded after five days and remained in the box until they matured to the specified age. In contrast, another group of specimens was demolded after a day and moved to a standard curing room maintained at 20 ± 2 °C. The two distinct curing processes, at −3 °C and standard room temperature, are depicted in Figure 1a and Figure 1b, respectively.

### 2.4. Test Methods

#### 2.4.1. Compressive Strength Test

**Figure 2:**
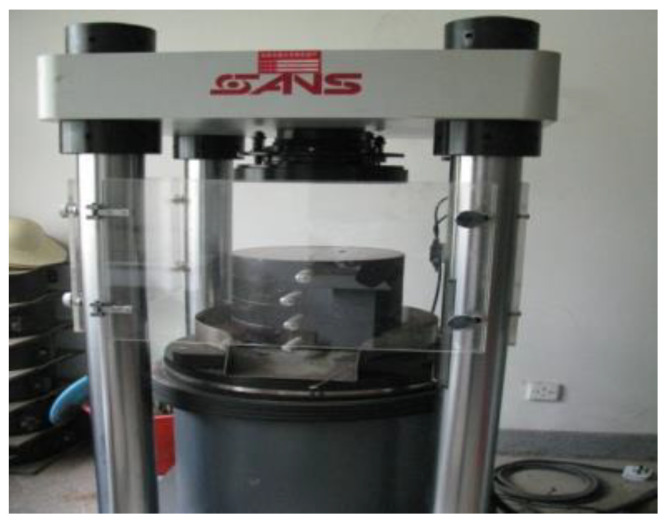
MTS electro-hydraulic servo press.

The concrete compressive strength test, conforming to the GB/T 50081–2019 standard [30], involved specimens sized 100 mm × 100 mm × 100 mm. These tests, conducted at 7, 14, 28, 56, 84, and 180 d using a pressure testing machine, assessed the strength of 432 specially prepared concrete blocks. The strength testing followed specified standards and was carried out with the MTS electro-hydraulic servo press (MTS systems Co., Ltd., Shanghai, China), as depicted in Figure 2.

#### 2.4.2. Freeze–Thaw Cycles (FTC) Test

**Figure 3:**
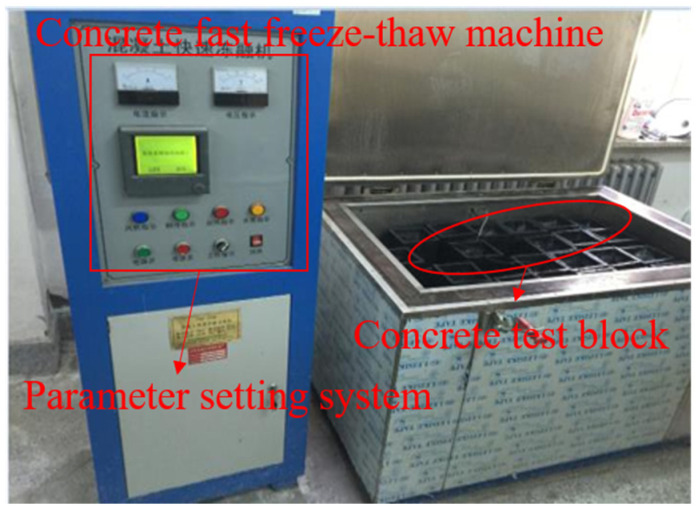
Concrete freeze–thaw circulator.

Concrete frost resistance tests were performed using the rapid freeze–thaw method as per the GB/T50082–2009 standard [31]. To assess pore structure, 216 cubes, each measuring 100 mm^3^, and 72 prismatic specimens of 100 mm × 100 mm × 400 mm were prepared. All procedures conformed to established standards. The FTC employed an automatic circulator, achieving freezing temperatures of −16 °C to −20 °C and melting temperatures of 3 °C to 7 °C, detailed in Figure 3 (Jian Yan Hua Ce science and technology Co., Ltd., Hangzhou, Zhejiang, China). The test protocol was as follows: Specimens underwent standard curing for 24 d, followed by 80 d at −3 °C, and then a 4-day water soak, ensuring uniform compressive strength. After soaking, their initial mass and dynamic modulus of elasticity (DME) were recorded. During the FTC test, measurements of transverse fundamental frequency were conducted every 25 cycles to assess external damage and mass loss. The mass loss rate (MLR) and DME were calculated using Equations (1) and (2), respectively. The relative dynamic modulus of elasticity (RDME) was computed with Equation (3) [31]. Frost resistance grade was determined by the maximum FTCs endured by a specimen, maintaining an MLR under 5% and an RDME above 60%.

(1) $$\Delta W_{N}=\frac{W_{0}-W_{N}}{W_{0}}\times 100\%,$$

where $\Delta W_{N}$ represents the MLR after N FTCs (%), $W_{0}$ is the initial mass (kg), and $W_{N}$ denotes the mass after N FTCs (kg).

(2) $$E=13.224\times 10^{-4}\times Wl^{3}f^{2}/a^{4},$$

where E is the DME (MPa), W is the mass (kg), accurate to 0.01 kg, l is the length (mm), f is the transverse fundamental frequency value (Hz), and a is the length of the cross-sectional side (mm).

(3) $$P_{N}=\frac{E_{N}}{E_{0}}\times 100\%=\frac{f_{N}^{2}}{f_{0}^{2}}\times 100\%,$$

where $P_{N}$ indicates the RDME after N FTCs (%), $E_{0}$ is the initial DME (MPa), $E_{N}$ denotes the DME after N FTCs (MPa), and $f_{0}$ and $f_{N}$ denote the initial transverse fundamental frequency before and after the freeze–thaw test (Hz), respectively.

#### 2.4.3. Pore Structure Testing Test

Cube specimens, cured at both standard and −3 °C conditions, were subjected to nuclear magnetic resonance (NMR) testing after 0, 50, and 100 FTCs. To fully saturate the internal pores, a 24-h vacuum and water saturation process was applied. This involved 3 h of dry pumping, 1 h of wet pumping following water injection, and a subsequent 20-h static phase for complete saturation. The vacuum, initially set at 0 MPa, reached −0.085 MPa within 2 min at a rate of 2 L/s, and −0.098 MPa by the 4 min mark. Following this, the specimens were immediately sealed in plastic to avoid dehydration. The experiment utilized the Suzhou Newmark Macro MR12–150H–I low-field magnetic resonance imaging machine (Suzhou, Jiangsu, China).

The pore characterization test utilized the NMR relaxation time method. It measured the transverse relaxation time ( $T_{2}$ ) of hydrogen atoms in water-saturated specimens, a key indicator of the pore size distribution in concrete [25]. The equation for calculating $T_{2}$ values in concrete’s water-bearing pores is detailed in Equation (4) [32].

(4) $$\frac{1}{T_{2}}=\rho_{2}\frac{S}{V},$$

where $\rho_{2}$ indicates the concrete surface relaxation rate and S/V is the specific surface area of the pores; for spherical pores or columnar pores, Equation (4) could be further converted to $T_{2}$ versus pore radius as shown in Equation (5) [32]:

(5) $$\frac{1}{T_{2}}=F_{S}\frac{\rho_{2}}{r},$$

where r is the pore radius (μm), and $F_{S}$ denotes the pore shape factor (3 for spherical pores and 2 for columnar pores). In this study, the pore shape was approximated as columnar pores, and the relaxation rate $\rho_{2}$ was 5 μm/s [33]. Therefore, the relationship between r and *T*_2_ can be reduced to Equation (6):

(6) $$r=0.01T_{2}.$$

## 3. Results and Discussion

### 3.1. Compressive Strength Analysis

**Figure 4:**
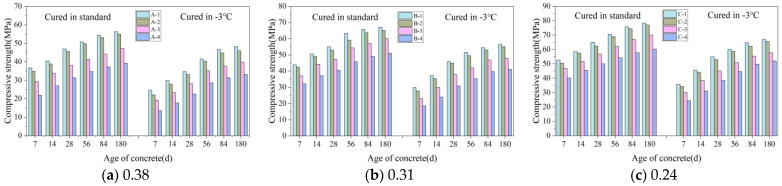
Variation of CSC with age for different w/b: (**a**) 0.38, (**b**) 0.31, and (**c**) 0.24.

Figure 4a–c show the variation in the CSC with age under different curing conditions when the w/b was 0.38, 0.31, and 0.24, respectively.

#### 3.1.1. Effect of Gas Content on Compressive Strength of Concrete (CSC)

Figure 4a shows that the A-1 specimen achieved a compressive strength of 34.7 MPa after 28 d at −3 °C. Compared to A-1, the compressive strengths of A-2, A-3, and A-4 were 95%, 81%, and 65%, respectively, indicating a decrease in CSC as gas content increased. This pattern, consistent across various w/b as shown in Figure 4a–c, reveals the impact of gas content on concrete strength. Higher gas content, especially in samples with identical mix ratios, resulted in more uniform spherical bubbles and increased porosity. This heightened porosity reduced the concrete’s load-bearing capacity, consequently lowering its strength. As the bubble count increased, a significant reduction in compressive strength was observed.

#### 3.1.2. Effect of Age on CSC

Figure 4a–c demonstrate that the CSC for all w/b improved over time. As an example, the CSC of the A-1 specimen, after 7 d of curing at −3 °C, was 24.6 MPa. This strength increased to 29.8 MPa, 34.7 MPa, 41.5 MPa, 47.2 MPa, and 52.5 MPa after 14, 28, 56, 84, and 180 d, respectively. These increases are attributed to the concrete’s ongoing hydration, which involves the formation of minerals like calcite and silicate. These minerals contribute to the concrete’s increased compactness and strength by filling its pores [34].

#### 3.1.3. Effect of w/b on the CSC

Maintaining constant gas content and age, a lower w/b in the concrete corresponded with increased compressive strength. For instance, the A-1, B-1, and C-1 specimens, all cured at −3 °C for 28 d, exhibited compressive strengths of 34.7 MPa, 45.9 MPa, and 54.9 MPa, respectively. The reduced w/b led to a higher concentration of cementitious materials, which accelerated their chemical reactions and crystallization. Furthermore, it diminished the gaps between cementitious material particles, enhancing their contact area. These factors collectively elevated the CSC [35].

#### 3.1.4. Effect of Curing Conditions on the CSC

When cured at −3 °C, the A-1 specimen’s compressive strength at 7, 14, 28, 56, 84, and 180 d showed reductions of 12.1 MPa, 10.7 MPa, 12.3 MPa, 9.3 MPa, 9.2 MPa, and 8.8 MPa, respectively, compared to standard curing conditions. Similar trends of decreasing strength over these durations were noted in other specimens, as illustrated in Figure 4a–c.

Concrete cured at −3 °C for 84 d showed compressive strength between 97% and 100% of that obtained from standard 28 d curing. With constant gas content, the CSC for concrete standardly cured for 28 d was similar to, or the same as, that of concrete cured for 84 d at −3 °C. This suggests that −3 °C curing takes longer to achieve the strength of standard curing due to the age-lag effect seen in negative-temperature conditions [36]. At −3 °C, the slower cement hydration leads to fewer hydration products and an increase in both unfilled pores and unhydrated cement, thus lowering CSC. Over time, however, the continued hydration process gradually enhances CSC.

### 3.2. FTC 

The specimens cured for 28 d under standard conditions and those cured for 84 d at −3 °C, with equivalent CSC, were selected for the FTC test. This choice enabled a direct comparison of the FRC under uniform CSC conditions [37].

#### 3.2.1. Apparent Concrete Damage and Degradation Characteristics

**Figure 5:**
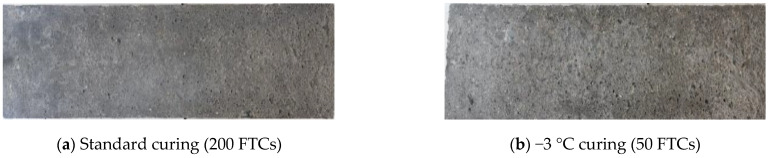
Appearance damage of A-2 specimen after FTC under different curing conditions.

Figure 5a shows that after undergoing 200 FTCs, the A-2 specimens under standard curing conditions displayed only slight mortar peeling and small pores on the surface; Figure 5b shows the surface damage of A-2 specimens after 50 FTCs under −3 °C curing condition. In contrast, when these specimens were subjected to −3 °C curing for only 50 FTCs, they experienced more pronounced surface mortar spalling than those under standard curing. Additionally, the pores on the surface of the concrete enlarged and became more numerous. This pattern indicates that the FRC is significantly lower with continuous curing at −3 °C compared to standard curing conditions.

**Figure 6:**
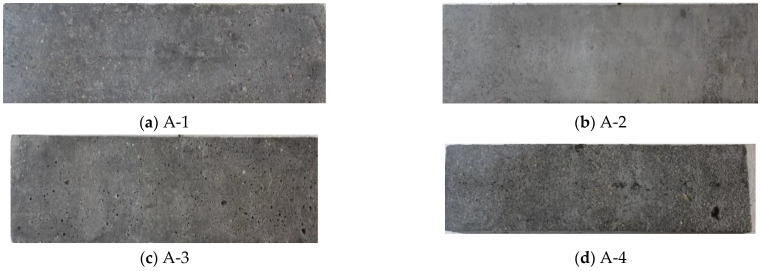
Appearance damage of concrete with different gas content after 150 FTCs under standard curing.

Figure 6 illustrates the damage on A-1 to A-4 specimens following 150 FTCs under standard curing. Minor cement mortar peeling is visible on the A-1 and A-2 specimens (Figure 6a,b), with A-2 showing less damage than A-1. The A-3 specimen (Figure 6c) presents more significant surface spalling and larger pores. The A-4 specimen (Figure 6d) exhibits severe damage, including extensive mortar peeling, larger pits, and exposed aggregate, indicating imminent failure. These findings indicate that the FRC improves up to a certain gas content level; however, an increase beyond this level leads to a sharp decrease in FRC.

**Figure 7:**
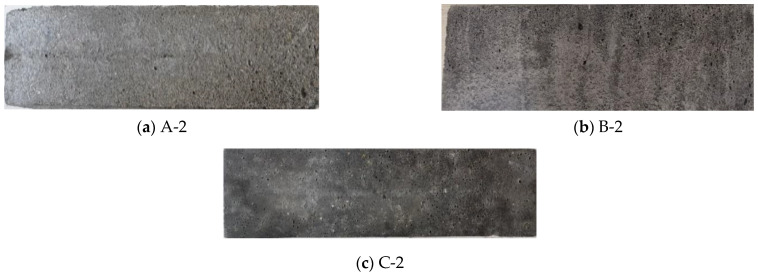
Appearance damage of concrete with different w/b after 250 FTCs under standard curing.

Figure 7 displays the damage to A-2, B-2, and C-2 specimens after undergoing 150 FTCs under standard curing. Figure 7a shows the A-2 specimen with significant surface damage, including extensive mortar peeling and visible holes. The B-2 specimen, as seen in Figure 7b, also experienced damage, although less severe than A-2. Conversely, the C-2 specimen, depicted in Figure 7c, showed only minor mortar peeling, indicating the least damage among the three. These findings imply that FRC is better with a lower w/b.

#### 3.2.2. Mass Loss Rate (MLR)

**Figure 8:**
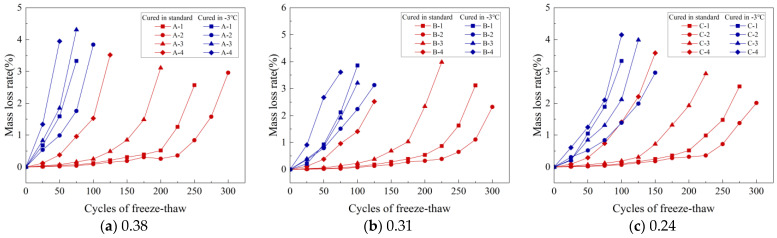
MLRs of concrete with different w/b after FTC: (**a**) 0.38, (**b**) 0.31, and (**c**) 0.24.

Figure 8a–c display how the MLR changes with the number of FTCs for specimens cured under standard conditions and at −3 °C for three different w/b: 0.38, 0.31, and 0.24. Notably, the MLRs for all specimens stayed below 5%. For example, the A-1 specimen recorded an MLR of 2.57% after 250 FTCs under standard curing. However, its RDME was only 47.26%, which is below the 60% threshold, indicating potential damage. Therefore, evaluating both MLR and RDME is crucial to determine the integrity of a concrete specimen accurately.

Figure 8a illustrates that the A-1 to A-4 specimens, when cured at −3 °C, experienced a rapid increase in MLRs after 25 FTCs, with the rates being 0.68%, 0.54%, 0.83%, and 1.34%, respectively. In comparison, specimens under standard curing showed a more modest increase in MLRs, ranging from 0.02% to 0.12% after the same number of FTCs. Similar trends were noted in concretes with various w/b. These findings imply that FRC tends to be higher under standard curing conditions compared to those at −3 °C.

Figure 8b displays the MLRs of B-1 to B-4 specimens after undergoing 100 FTCs under standard curing, with MLRs recorded at 0.11%, 0.08%, 0.23%, and 1.41%, respectively. In comparison, the same specimens subjected to 50 FTCs at −3 °C had MLRs of 0.93%, 0.79%, 0.86%, and 2.67%. Notably, the B-2 specimen showed the lowest MLR under both curing conditions at these cycle counts, indicating its enhanced frost resistance. This trend suggests that the FRC increases within a certain gas content range. Similar trends were also observed in concretes with different w/b, as demonstrated in Figure 8a,c.

Figure 8a–c illustrate that under −3 °C curing, the MLRs for A-2, B-2, and C-2 after 100 FTCs were 3.84%, 2.24%, and 1.39%, respectively. A lower w/b leads to a reduced MLR, indicating improved FRC as the w/b ratio decreases.

#### 3.2.3. Relative Dynamic Modulus of Elasticity (RDME)

**Figure 9:**
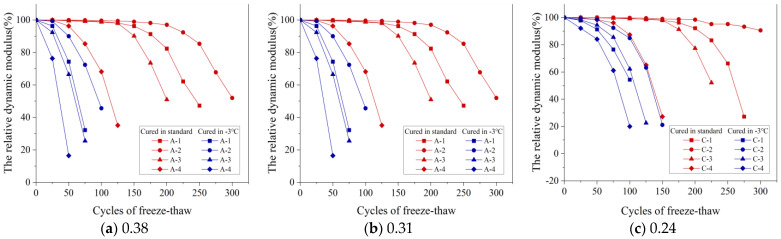
RDMEs of concrete with different w/b after FTC: (**a**) 0.38, (**b**) 0.31, and (**c**) 0.24.

Figure 9a–c display how the RDME varies with the number of FTCs for concrete mixtures with three w/b: 0.38, 0.31, and 0.24. These were tested under standard conditions and at −3 °C. Specifically, Figure 9a shows a marked decrease in RDME after 25 FTCs at −3 °C, with specimen A–1′s RDME falling to 32.19% after 75 FTCs. In stark contrast, under standard curing conditions, A–1′s RDME significantly dropped only after 175 FTCs, landing at 47.26% after 250 FTCs. The FRC under −3 °C curing was notably lower than that under standard curing, primarily due to extensive micro-cracks in the concrete at negative temperatures. These cracks propagate rapidly during FTCs, heightening the risk of denudation and, therefore, reducing FRC. This pattern in RDME was similarly observed across other w/b.

As the gas content in concrete rises, its ability to endure FTCs initially increases but then decreases. This occurs because, within an optimal gas content range, the concrete’s internal pore structure is enhanced, reducing the ice heave and capillary water osmosis pressures during freeze–thaw processes and thus improving the FRC. However, exceeding this optimal range leads to an overabundance of gas, causing internal bubbles to interconnect with pores. This weakens the concrete’s internal structure under freezing and thawing conditions, therefore reducing FRC.

In this study, the FRC was evaluated through its appearance, MLR, and RDME. Optimal FRC was observed when the gas content ranged between 3.2% and 3.8%.

#### 3.2.4. Microscopic Pore Structure

##### Porosity

**Figure 10:**
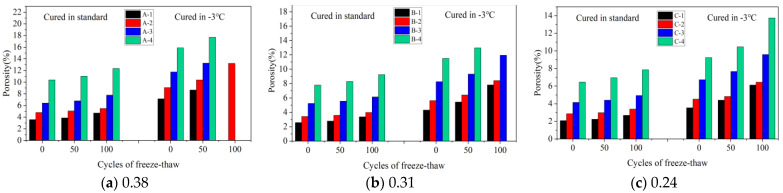
Porosity of concrete with different w/b after FTCs: (**a**) 0.38, (**b**) 0.31, and (**c**) 0.24.

Porosity is an important index that reflects the microscopic properties of concrete. Figure 10a–c show the variation in porosity with the number of FTCs for three w/b (0.38, 0.31, and 0.24) of concrete under standard and −3 °C curing, respectively. Specimens A-1, A-3, A-4, and B-4, which were cured at −3 °C, were destroyed after 100 FTCs. As shown in Figure 10a, for specimens A-1–A-4, the porosity of concrete under standard curing and without FTC was 3.58%, 4.79%, 6.42%, and 10.38%, respectively. The porosities of concrete after 100 FTCs were 4.71%, 5.48%, 7.78%, and 12.36%, respectively. These values increased by 1.13%, 0.69%, 1.36%, and 1.98%, respectively, compared with the specimens without FTCs. The porosity of concrete increased with the increase in the number of FTCs, and the porosity of the A-2 specimen increased the least. This indicates that the gas content can improve the FRC within a certain range. This was consistent with the results of the macro analysis. 

Figure 10a–c illustrate the changes in porosity, a key indicator of concrete’s microscopic properties, across a range of FTCs for concrete with three w/b (0.38, 0.31, and 0.24), under both standard and −3 °C curing conditions. Specimens A-1, A-3, A-4, and B-4, cured at −3 °C, experienced destruction after 100 FTCs. Figure 10a shows that initially, under standard curing and without FTCs, the porosity for specimens A-1 to A-4 was 3.58%, 4.79%, 6.42%, and 10.38%, respectively. After undergoing 100 FTCs, these percentages increased to 4.71%, 5.48%, 7.78%, and 12.36%, respectively, with rises of 1.13%, 0.69%, 1.36%, and 1.98%. This pattern reveals that porosity increases with more FTCs, and notably, specimen A-2 exhibited the smallest increase. These observations indicate that gas content within a certain range can improve the FRC, corroborating the results of macro-level analysis.

Figure 10a presents the porosity changes in specimen A-2 under standard and −3 °C curing. Initially, porosity under standard curing was 4.79%, rising to 5.06% and 5.48% after 50 and 100 FTCs, respectively. Conversely, at −3 °C, the porosity started at 9.08% and increased to 10.39% and 13.23% after 50 and 100 FTCs, showing respective increases of 4.29%, 5.33%, and 7.75% compared to standard curing. These results highlight a significant rise in porosity with increased FTCs under −3 °C, more so than under standard conditions, indicating a pronounced effect of lower temperatures on concrete porosity.

Figure 10a–c demonstrate that initially, without FTCs and under standard curing, specimens A-2, B-2, and C-2 exhibited porosities of 4.79%, 3.42%, and 2.87%, respectively. After 100 FTCs, these values increased to 5.48%, 3.98%, and 3.40%. Importantly, it was observed that a lower w/b resulted in a gradual decrease in the porosity of the concrete.

##### Percentage of Pore Size Distribution

**Figure 11:**
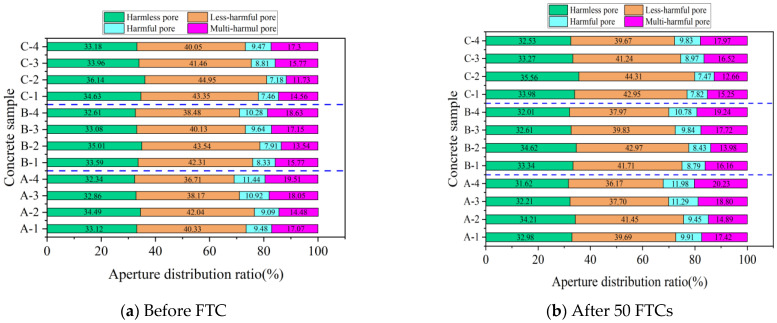
NMR pore size distribution of concrete before and after 50 FTCs under standard curing.

**Figure 12:**
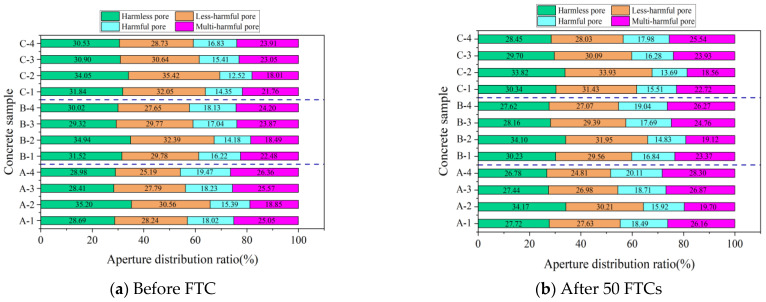
NMR pore size distribution of concrete before and after 50 FTCs under −3 °C curing.

According to Wu [38], concrete pores are classified into four categories: harmless (r < 20 nm), less harmful (20 nm ≤ r < 100 nm), harmful (100 nm ≤ r ≤ 200 nm), and multi-harmful (r > 200 nm). Figure 11 and Figure 12 show the NMR analysis of pore size distribution in concrete subjected to 50 FTCs under both standard and −3 °C curing. These figures illustrate a clear pattern: with an increasing number of FTCs, the proportion of harmless and less harmful pores decreases, while harmful and multi-harmful pores increase for both curing conditions. This trend is more evident under −3 °C curing, suggesting reduced FRC in comparison to standard curing conditions.

Figure 11a presents the pore distribution in concrete specimens A-1 through A-4, which had not undergone FTCs and were subjected to standard curing. In descending order, the specimens with the highest proportion of harmless and less harmful pores were A-2, followed by A-1, A-3, and A-4. This ordering is also observed in concretes with different w/b. The pattern is attributed to the inclusion of small, uniformly distributed bubbles created by the air-entraining agent. A well-calibrated amount of these bubbles can significantly reduce the presence of harmful and multi-harmful pores, thus improving the FRC [15].

Figure 12a reveals that under −3 °C curing and without undergoing FTCs, the percentages of harmless pores in specimens A-1, B-1, and C-1 were 28.69%, 31.52%, and 31.84%, respectively. Concurrently, the less harmful pores accounted for 28.24%, 29.78%, and 32.05%. A notable trend emerged: a decrease in the w/b led to an increase in the percentage of harmless and less harmful pores while simultaneously decreasing the percentages of harmful and multi-harmful pores. This trend contributes to an improvement in the FRC, a conclusion that is consistent with earlier analyses of macroscopic performance indices and porosity.

## 4. Evolutionary Analysis of Freeze–Thaw Damage (FTD)

### 4.1. Analysis of FTD Process

To describe the damage process of concrete, the DME was used to represent the damage variable according to damage mechanics theory, and the corresponding macroscopic damage evolution equation of concrete was established [39]. This equation is shown in Equation (7).

(7) $$D=1-P_{N},$$

where D is the FTD variable.

**Figure 13:**
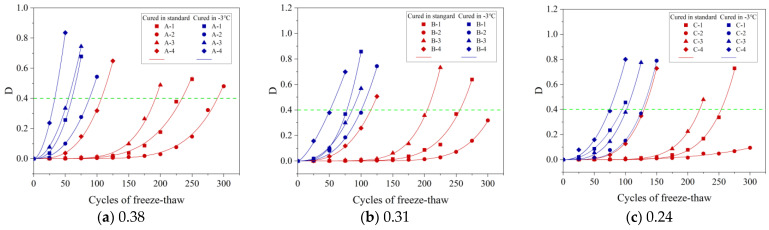
FTD variables of concrete with different w/b. D is the FTD variable expressed by the RDME.

According to Figure 13, D is the FTD variable, the D increased with the increase in the number of FTCs. Therefore, the power function can be used to establish the relationship between FTD variables and FTCs of different concrete specimens [40], as shown in Equation (8).

(8) $$D=a\times N^{b},$$

where N is the number of FTCs, and a and b are the fitting coefficients.

Table 4 presents the fitting coefficients, which illustrate the relationship between concrete’s FTD variable and the quantity of FTCs. The correlation coefficients (R^2^) for the FTD variables are predominantly above 0.97, often surpassing 0.99. This high level of R^2^ values demonstrates the power function’s accuracy in modeling this relationship. The FTD model, referenced in studies by Yu et al. [24] and Bai et al. [25], includes both the count of FTCs and the concept of freeze–thaw fatigue life, which is the maximum number of FTCs a concrete can endure. This approach enables the use of power function fitting results to accurately estimate the freeze–thaw fatigue life of concrete.

### 4.2. Freeze–Thaw Fatigue Life

The GB/T 50082–2009 standard [31] sets the damage threshold for concrete in underwater freeze–thaw conditions as a 40% reduction in RDME, denoted as FTD variable D = 0.4. This benchmark is crucial for calculating the freeze–thaw fatigue life of concrete, as outlined in Equation (8). Table 5 shows this fatigue life as the highest number of FTCs concrete withstands before failure. Notably, under standard curing, an A-2 specimen endured 292 FTCs but only 88 FTCs at −3 °C, indicating longer fatigue life at standard conditions. For the B-series specimens under standard curing, their fatigue lives (B-1 to B-4) were 256, 310, 204, and 115 FTCs, respectively, suggesting that increased gas content initially enhances, then decreases, FTC tolerance. Conversely, under −3 °C curing, A-1, B-1, and C-1 specimens lasted for 60, 77, and 94 FTCs, respectively, highlighting that lower w/b ratios lengthen fatigue life. These results refine the FTD model, correlating the FTD variable with the maximum FTCs for different curing conditions.

### 4.3. FTD Model

#### 4.3.1. FTD Model Based on RDME

Yu et al. established an FTD model for concrete based on the fatigue damage accumulation theory [24,41], as shown in Equations (9) and (10).

(9) $$D=1-\frac{E(N)}{E_{0}}=1-\frac{1-\beta \log N_{f}}{1-\beta \log(N_{f}-N)},$$

(10) $$\beta =\frac{0.4}{\log N_{f}},$$

where D = 0.4 is the critical condition value for FTD of concrete [30] and β is the material parameter.

According to the principles of irreversible thermodynamics and continuous damage mechanics, Bai et al. proposed the FTD model of concrete by introducing the fatigue damage theory [25,41,42], as shown in Equation (11).

(11) $$D=D_{c}\left[1-{\left(1-\frac{N}{N_{f}}\right)}^{\frac{1}{2k+2}}\right],$$

where $D_{c}$ was the critical damage factor for FTD obtained from the damage variable. In this study, the RDME was used as the damage variable, so the critical damage factor $D_{c}$ = 0.4 and k denotes a material parameter.

**Figure 14:**
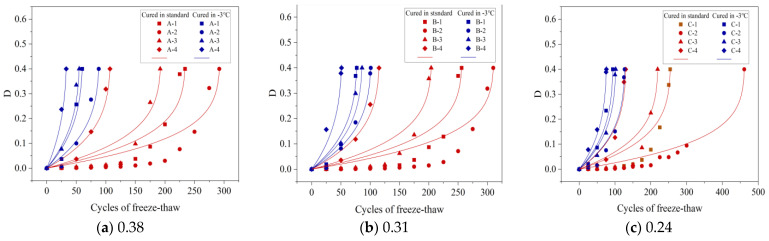
Relationship curves between FTD variables and the number of FTCs for concrete with different w/b in the Yu model.

**Figure 15:**
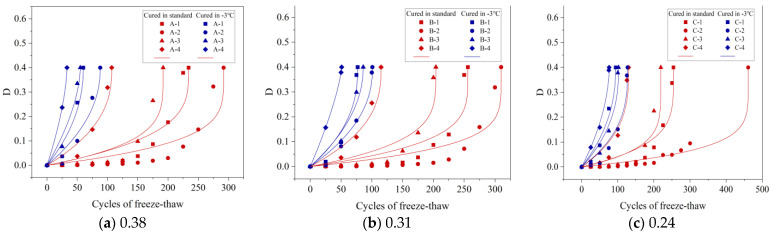
Relationship curves between FTD variables and the number of FTCs for concrete with different w/b in the Bai model.

In this research, the models established by Yu and Bai were utilized to define the correlation between the FTD variable and the quantity of FTCs experienced by concrete. This relationship is visually represented in Figure 14 and Figure 15 through fitted curve plots. Detailed descriptions of each curve’s parameters and their respective correlation coefficients are presented in Table 6 and Table 7. The findings demonstrate a close alignment between the models and the experimental results, with both models showing high correlation coefficients. In particular, these models more accurately depicted the relationship of the FTD variable with the number of FTCs under −3 °C curing conditions, achieving correlation coefficients (R^2^) above 0.95. This high correlation underscores the effectiveness of these models in predicting the FTD degradation of concrete under −3 °C curing scenarios. Additionally, the Bai model, which exhibited a slightly higher correlation coefficient than the Yu model, was found to predict the progression of damage more precisely in concrete subjected to −3 °C curing conditions.

#### 4.3.2. FTD Model Parameters

**Figure 16:**
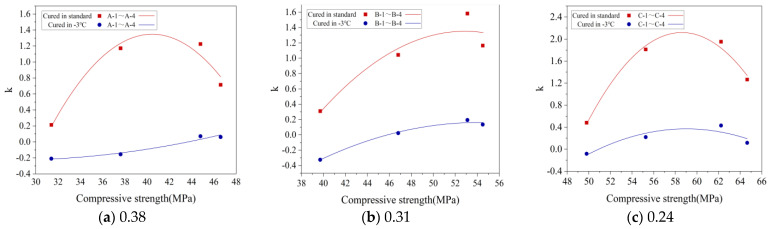
Fitting relationship curve between material parameter *k* and compressive strength in the Bai model. *k* denotes a material parameter.

Section 3.1.1 clearly establishes that the CSC decreases as gas content increases, highlighting a direct relationship between the two. This relationship supports the development of FTD models that factor in CSC to evaluate the effect of gas content on FTD. In Section 4.3.1, the Bai model is identified as superior to the Yu model for accurately predicting the progression of concrete damage. As such, a quadratic polynomial fitting approach is apt for correlating the Bai model’s material parameter k with CSC. This correlation is visually represented in Figure 16, where the fitting curve between material parameter k and CSC is displayed. Table 8 provides the detailed fitting equation and correlation coefficient, confirming the model’s precise fitting capabilities for different groups.

#### 4.3.3. FTD Model for Concrete under Different Curing Conditions

The Bai model’s fitting formula for the material parameter k, in correlation with the CSC, has been incorporated into Equation (11) to develop an FTD model. This model accounts for the 28-day standard curing compressive strength across different curing scenarios. It facilitates predicting concrete’s FTD level under varying curing conditions by referencing the 28-day standard curing strength. One of the key benefits of this model is that it bypasses the need for individual material parameter adjustments for each concrete sample. This efficiency makes the model exceptionally valuable for technical assessments of FRC, especially in permafrost areas.

Taking specimens A-1 to A-4 as an example, the FTD model based on the Bai model is shown in Equation (12).

(12) $$D=0.4\times \left[1-{\left(1-\frac{N}{N_{f}}\right)}^{\frac{1}{2\times \left(0.008439\times f_{c}^{2}-0.04584\times f_{c}+0.39239\right)+2}}\right]$$

## 5. Life Expectancy Projection

Concrete’s durability in actual environments varies due to numerous factors, resulting in different lifespans under diverse conditions. Section 4.2 explores concrete’s freeze–thaw fatigue life, defined by the maximum FTCs it endures. However, realistically, concrete’s lifespan should be measured in years [43]. Li et al. [44] bridged the gap between lab test data and real-world freeze–thaw durability. They formulated an equation linking indoor and outdoor FTCs across various regions, considering water-induced freeze–thaw effects. This correlation is detailed in Equation (13) [43].

(13) $$T=\frac{eN_{f}}{M},$$

where T is the actual service life of the concrete (years), and e is the FTC coefficient, which is the ratio of one indoor FTC to the number of outdoor natural FTCs. In this study, we used the value of 12 [43]. M indicates the number of FTCs that the concrete undergoes in 1 year in the actual environment.

The annual average number of FTCs in Northwest China could be known from [45], as shown in Table 9. The service life of concrete in Northwest China can be obtained by substituting the test data of existing concrete into Equation (13). This is shown in Table 10.

Table 9 indicates a marked contrast in the durability of concrete under different curing conditions. Although concrete cured under standard conditions shows a potential lifespan of up to 46.9 years, the same concrete cured at −3 °C only lasts up to 12.9 years, highlighting a significant decrease in durability due to lower curing temperatures. For concrete specimens A-1 through A-4, cured under standard conditions, their lifespans were 23.8, 29.7, 19.5, and 10.9 years, respectively, with A-2 outlasting the others. It is also evident that concrete achieves its maximum longevity within a gas content range of 3.2% to 3.8%. Furthermore, a lower w/b is associated with longer concrete lifespans.

## 6. Conclusions

In this research, we focused on the strength and FRC in concrete cured at −3 °C. Through NMR testing, we investigated the concrete’s pore structure before and after experiencing FTCs. Our key conclusions are:

(1) Increasing gas content in concrete correlates with a reduction in its CSC. This reduction is mainly due to the rise in air bubbles, diminishing the concrete’s load-bearing capacity. Moreover, the CSC is substantially affected by curing conditions. For instance, at various ages under −3 °C curing, the CSC was only 57.8% to 86.4% of what was observed under standard conditions, displaying a significant age-lag phenomenon. This is likely due to the slowed cement hydration reaction at lower temperatures, resulting in fewer hydration products and reduced compactness.
(2) The optimal FRC was noted when the gas content was between 3.2% and 3.8%. After 50 FTCs, the RDME of A-1 specimens cured at standard conditions for 28 d and −3 °C for 84 d was 99.76% and 74.32%, respectively. This shows that concrete experiences more severe initial damage under −3 °C curing compared to standard conditions. Despite similar CSC, FRC at −3 °C is significantly lower, primarily due to micro-cracks that rapidly expand during FTCs, increasing vulnerability to damage.
(3) Concrete porosity tends to increase with the number of FTCs. The maximum number of harmless and less harmful pores was observed in concrete with a gas content ranging from 3.2% to 3.8%. Notably, the porosity under −3 °C curing was much higher than under standard conditions, affirming the frost resistance findings from both macroscopic and microscopic analyses.
(4) We developed an FTD model tailored for concrete cured at −3 °C, using results from freeze–thaw tests and existing models. This model accurately predicts FTD in concrete after 28 days of standard curing, which is vital for assessing FTD in permafrost areas.
(5) In general, concrete has its longest lifespan when the gas content is between 3.2% and 3.8%. Under standard conditions, concrete’s lifespan can reach up to 46.9 years, but it drops to just 12.9 years under −3 °C curing. This highlights the need for careful construction and curing practices in cold environments.

Although this study provides insights into the CSC and FRC of concrete under −3 °C and standard curing conditions, the real-world complexity of permafrost regions warrants further study. More comprehensive research is needed to fully understand the effects of different temperature curing on the mechanical properties and longevity of concrete.

## Figures and Tables

**Table 1:** Technical specifications of the cement.

Cement Variety	Specific Surface Area (m^2^/kg)	Coagulation Time (min)		Flexural Strength (MPa)		Compressive Strength (MPa)	
		Initial	Final	3 d	28 d	3 d	28 d
P.O 42.5	360	95	140	5.5	8.9	23.4	46.6

**Table 2:** Fine aggregate particle grading.

Mesh Size (mm)	Sub-Count Screening Residue Rate (%)	Cumulative Screening Rate (%)	Particle Gradation Zone Genus	Fineness Modulus
4.75	8.7	8.7	III	2.2
2.36	7.3	16.0		
1.18	6.5	22.5		
0.60	13.3	35.8		
0.30	35.5	71.3		
0.15	24.4	95.7		

**Table 3:** Concrete mix proportions.

Water–Binder Ratio (w/b)	Specimen Number	Water (kg/m^3^)	Cement (kg/m^3^)	Mineral Powder (kg/m^3^)	Fly Ash (kg/m^3^)	Sand Rate (%)	Water-Reducing Agent (%)	Air-Entraining Agent (%)	Gas Content (%)
0.38	A-1	150	276	59	59	46	1.5	0	1.5
	A-2	150	276	59	59	46	1.5	0.03	3.8
	A-3	150	276	59	59	46	1.5	0.06	6.4
	A-4	150	276	59	59	46	1.5	0.09	9.6
0.31	B-1	144	325	70	70	44	1.9	0	1.4
	B-2	144	325	70	70	44	1.9	0.02	3.5
	B-3	144	325	70	70	44	1.9	0.04	5.8
	B-4	144	325	70	70	44	1.9	0.06	8.1
0.24	C-1	140	408	88	88	42	3.5	0	0.8
	C-2	140	408	88	88	42	3.5	0.02	3.2
	C-3	140	408	88	88	42	3.5	0.04	5.5
	C-4	140	408	88	88	42	3.5	0.06	8.4

**Table 4:** Fitted coefficients in the power function.

Conservation Conditions	Specimen Number	a	b	R^2^
Standard curing	A-1	3.337 × 10^−12^	4.676	0.990
	A-2	4.341 × 10^−16^	6.077	0.994
	A-3	3.209 × 10^−13^	5.297	0.995
	A-4	2.597 × 10^−7^	3.050	0.999
−3 °C curing	A-1	1.772 × 10^−5^	2.444	0.990
	A-2	7.535 × 10^−6^	2.430	0.999
	A-3	1.314 × 10^−4^	2.002	1.000
	A-4	6.785 × 10^−4^	1.819	1.000
Standard curing	B-1	9.468 × 10^−17^	6.489	0.994
	B-2	1.821 × 10^−20^	7.766	0.999
	B-3	1.335 × 10^−15^	6.266	0.999
	B-4	3.727 × 10^−7^	2.924	1.000
−3 °C curing	B-1	8.683 × 10^−7^	2.998	1.000
	B-2	1.165 × 10^−6^	2.766	0.998
	B-3	1.039 × 10^−5^	2.370	0.999
	B-4	1.490 × 10^−3^	1.424	0.999
Standard curing	C-1	2.779 × 10^−18^	7.137	0.998
	C-2	3.857 × 10^−10^	3.385	0.979
	C-3	2.071 × 10^−16^	6.531	0.998
	C-4	6.477 × 10^−10^	4.160	1.000
−3 °C curing	C-1	9.437 × 10^−6^	2.343	1.000
	C-2	1.557 × 10^−9^	3.999	0.998
	C-3	1.566 × 10^−7^	3.192	0.999
	C-4	1.783 × 10^−5^	2.324	0.994

**Table 5:** Freeze–thaw fatigue life of concrete.

Conservation Conditions	Standard Curing				−3 °C Curing			
Specimen number	A-1	A-2	A-3	A-4	A-1	A-2	A-3	A-4
$N_{f}$	234	292	192	107	60	88	55	33
Specimen number	B-1	B-2	B-3	B-4	B-1	B-2	B-3	B-4
$N_{f}$	256	310	204	115	77	101	86	51
Specimen number	C-1	C-2	C-3	C-4	C-1	C-2	C-3	C-4
$N_{f}$	254	461	219	130	94	127	102	76

**Table 6:** Fitting parameters and correlation coefficients in the Yu model.

Conservation Conditions	Specimen Number	$N_{f}$	β	R^2^
Standard curing	A-1	234	0.169	0.878
	A-2	292	0.162	0.827
	A-3	192	0.175	0.861
	A-4	107	0.197	0.964
−3 °C curing	A-1	60	0.225	0.969
	A-2	88	0.206	0.957
	A-3	55	0.230	0.963
	A-4	33	0.263	0.984
Standard curing	B-1	256	0.166	0.843
	B-2	310	0.161	0.790
	B-3	204	0.173	0.873
	B-4	115	0.194	0.970
−3 °C curing	B-1	77	0.212	0.974
	B-2	101	0.200	0.988
	B-3	86	0.207	0.951
	B-4	51	0.234	0.966
Standard curing	C-1	254	0.166	0.864
	C-2	461	0.150	0.946
	C-3	219	0.171	0.809
	C-4	130	0.189	0.937
−3 °C curing	C-1	94	0.203	0.974
	C-2	127	0.190	0.971
	C-3	102	0.199	0.981
	C-4	76	0.213	0.989

**Table 7:** Fitting parameters and correlation coefficients in the Bai model.

Conservation Conditions	Specimen Number	$N_{f}$	k	R^2^
Standard curing	A-1	234	0.715	0.859
	A-2	292	1.225	0.849
	A-3	192	1.172	0.893
	A-4	107	0.213	0.953
−3 °C curing	A-1	60	0.061	0.966
	A-2	88	0.071	0.959
	A-3	55	−0.155	0.974
	A-4	33	−0.210	1.000
Standard curing	B-1	256	1.165	0.862
	B-2	310	1.583	0.858
	B-3	204	1.044	0.889
	B-4	115	0.310	0.964
−3 °C curing	B-1	77	0.194	0.955
	B-2	101	0.135	0.980
	B-3	86	0.023	0.956
	B-4	51	−0.324	1.000
Standard curing	C-1	254	1.266	0.981
	C-2	461	1.953	0.903
	C-3	219	1.812	0.910
	C-4	130	0.483	0.920
−3 °C curing	C-1	94	0.117	0.977
	C-2	127	0.432	0.955
	C-3	102	0.220	0.961
	C-4	76	−0.082	0.992

**Table 8:** Fitting equations and correlation coefficients for material parameter *k* and compressive strength ( $f_{c}$ ) in the Bai model.

Conservation Conditions	Specimen Number	The Relationship between *k* and *f_c_*	R^2^
Standard curing	A-1–A-4	$k=-0.01406\times f_{c}^{2}+1.13716\times f_{c}-21.65040$	0.948
−3 °C curing	A-1–A-4	$k=-0.00084\times f_{c}^{2}-0.04584\times f_{c}+0.39239$	0.959
Standard curing	B-1–B-4	$k=-0.00610\times f_{c}^{2}+0.64468\times f_{c}-15.68860$	0.893
−3 °C curing	B-1–B-4	$k=-0.00235\times f_{c}^{2}+0.25376\times f_{c}-6.70378$	0.987
Standard curing	C-1–C-4	$k=-0.02137\times f_{c}^{2}+2.50491\times f_{c}-71.27963$	0.982
−3 °C curing	C-1–C-4	$k=-0.00560\times f_{c}^{2}+0.66119\times f_{c}-19.13907$	0.814

**Table 9:** The average annual number of FTCs in Northwest China.

Area	Extreme Minimum Temperature (°C)	Average Annual Temperature Difference (°C)	Average Annual Low Temperature Days (d)	Average Annual FTCs (Times)
Northwest China	−26.2	52.1	169	118

**Table 10:** Life expectancy of concrete in Northwest China.

Conservation Conditions	Standard Curing				−3 °C Curing			
Specimen number	A-1	A-2	A-3	A-4	A-1	A-2	A-3	A-4
Service life (years)	23.8	29.7	19.5	10.9	6.1	8.9	5.6	3.4
Specimen number	B-1	B-2	B-3	B-4	B-1	B-2	B-3	B-4
Service life (years)	26	31.5	20.7	11.7	7.8	10.3	8.7	5.2
Specimen number	C-1	C-2	C-3	C-4	C-1	C-2	C-3	C-4
Service life (years)	25.8	46.9	22.3	13.2	9.6	12.9	10.4	7.7

## Data Availability

Data are contained within the article.
